# Evaluating maternal and child health indicators for the Sustainable Development Goals in 2018: what is Iran’s position?

**DOI:** 10.4178/epih.e2019045

**Published:** 2019-10-11

**Authors:** Elham Khatooni, Isa Akbarzadeh, Elham Abdalmaleki, Zhaleh Abdi, Elham Ahmadnezhad

**Affiliations:** 1 Department of Epidemiology and Biostatistics, School of Public Health, Tehran University of Medical Sciences, Tehran, Iran; 2 National Institute for Health Research, Tehran University of Medical Sciences, Tehran, Iran

**Keywords:** Maternal health, Child health, Sustainable development, Human development index, Iran

## Abstract

**OBJECTIVES:**

Since many Millennium Development Goals (MDGs) were not achieved, countries including Iran—despite achieving some of the MDGs—need regular planning to achieve the Sustainable Development Goals (SDGs) by 2030. This article examines maternal and child health indicators in the early years of the SDGs in Iran relative to several other countries.

**METHODS:**

This study was carried out through a secondary analysis of maternal and child health indicators in Iran. The results were compared with data from other countries divided into three groups: countries with upper-middle income levels, countries in the Eastern Mediterranean region, and the countries covered by the Outlook Document 1,404 (a regional classification). Then, the relationship between these indicators and the Human Development Index was investigated.

**RESULTS:**

Iran has attained better results than other countries with respect to maternal mortality, family planning, skilled birth attendance, under-5 deaths, incidence of hepatitis B, diphtheria-tetanus-pertussis vaccination coverage, and antenatal care. In contrast, Iran performed worse than other countries with respect to under-5 wasting, under-5 stunting, and care-seeking behavior for children.

**CONCLUSIONS:**

Overall, among the 11 indicators surveyed, Iran has attained better-than-average results and seems to be improving. We recommend that Iran continue interventions in the field of maternal and child health.

## INTRODUCTION

In 2015, the United Nations General Assembly, endeavoring to reach the Millennium Development Goals (MDGs) set 15 years earlier, adopted a plan termed “Transforming Our World: the 2030 Agenda for Sustainable Development,” which provides a framework for drawing up a Sustainable Development Plan by 2030. The program includes 17 goals that participating countries are expected to achieve by the year 2030 [1]. The MDGs ended in 2015 and did not achieve their aim of a 75% reduction in maternal mortality. In fact, most countries failed to achieve their maternal mortality reduction goals [2]. Because mothers’, infants’, and children’s health is important in societies at all stages of development, it is crucial to focus on maternal and child health in the context of achieving goals set for sustainable development [3,4].

One of the 17 Sustainable Development Goals (SDGs) focuses on the maintenance and promotion of the health of people of all ages (SDG 3). Goal 3.8 of SDG 3 refers to attaining universal health coverage [5]. The World Health Organization (WHO) has stated that universal health coverage means that “all people and communities can use the promotive, preventive, curative, rehabilitative, and palliative health services they need, of sufficient quality to be effective, while also ensuring that the use of these services does not expose the user to financial hardship” [6].

Iran has performed acceptably on MDGs related to maternal and child health; in the area of under-5 deaths, Iran achieved the MDG target by 2015, with a 70% reduction in the deaths of children under 5 years between 1990 and 2015 [7]. The maternal death rate declined by about 78% between 1990 and 2015, dropping from 123 deaths to 25 deaths per 100,000 live births [8]. However, the trends for under-5 overweight, wasting, and stunting in Iran have been non-uniform between 1998 and 2013 [7].

Several interventions can help improve maternal and child health, and the effects of these interventions can be assessed using specific indicators. However, a better view of the situation can sometimes be outlined by combined indicators, one of which is the Human Development Index (HDI). The HDI is an average indicator of overall achievement across three dimensions: lifespan, education, and standard of living. According to the latest report published by the UN, Iran ranks 60th among the 189 countries assessed, with a score of 0.798 [9]. Recent studies have stated that the HDI is not only a good indicator of a country’s socioeconomic development, but can also serve as a predictor of some health indicators, including maternal and infant death [10,11], pre-pregnancy and pregnancy outcomes [12], preterm delivery [13], quality of life associated with children’s health [14], and under-5 death [15]. Therefore, we examined the associations between the selected indicators in this study using the HDI.

By clarifying the status of Iran with regard to maternal and child health-related indicators, it is possible to move toward achieving the SDGs by 2030. Moreover, by clarifying Iran’s performance in targeted areas, the strengths and weaknesses of existing programs can be assessed, and more appropriate interventions can be developed. Therefore, this study aimed to assess maternal and child health indicators in Iran, to compare the results with those of countries in the region with a similar situation, and to provide an overview of Iran’s status compared with other countries.

## MATERIALS AND METHODS

### Data

For this ecological study, the most recently reported data for 11 relevant indicators were extracted from the SDG dashboard of the WHO (Table 1) [16] between January and March 2019, at the National Institute for Health Research. National populations and the world mean of each indicator were extracted from the World Bank website [17]. The HDI data were extracted from the Human Development Reports database [9].

### Variables, indicators, and analysis

#### Indicators

The definition of each indicator is given in Table 1.

These 11 indicators were selected as measures of maternal and child health status that have also been targeted by the SDGs.

#### Indicators’ weighted mean, total mean, median, and rankings

Since the index values provided by the databanks did not consider population, to provide a better comparison, weighted means were calculated for each indicator.

The following formula was used to calculate the mean of each indicator for each group of countries in order to compare the indicator in Iran with the total mean (Table 2): $\mathrm{Mean}\;\mathrm{indicator}\;\mathrm{for}\;\mathrm{each}\;\mathrm{region}\;=\frac{\Sigma population\;of\;country\;\times \;indicator}{\Sigma population\;of\;countries\;of\;group}$

When calculating the weighted mean of an indicator for a country within a certain group, if an indicator was not reported for a country, its population was not included in the denominator of the calculation. A reported indicator may appear more favorable in a country with a small population. Calculating the weighted mean provides values for each indicator that can be compared across countries, unlike the crude values.

The total mean was calculated by taking the mean of the values for all 75 countries without considering the weight (population) of each country. The global mean for each indicator was taken from the World Bank database [18].

**Figure 1.**
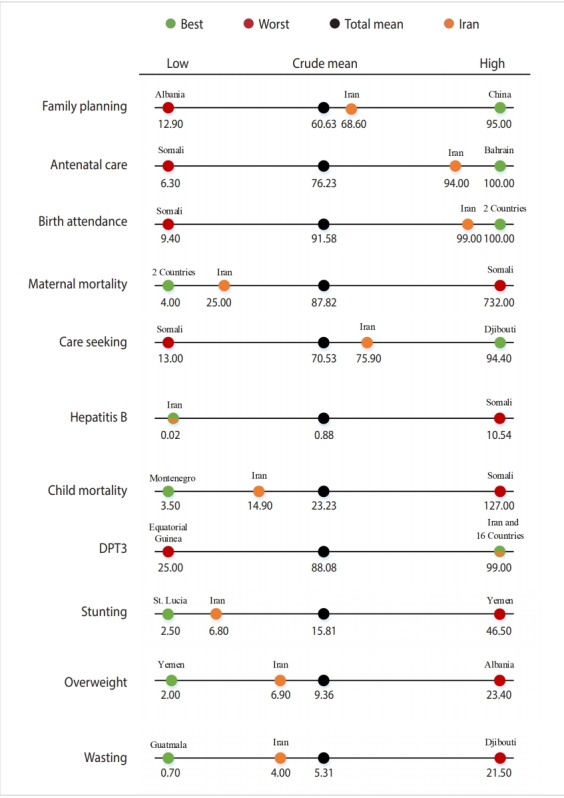
Iran rank in comparison with total (crude) mean, best and worst countries. DTP3, diphtheria-tetanus-pertussis.

Figure 1 shows the best-performing and worst-performing country for each indicator compared to the total mean and Iran’s position compared to the best-performing and worst-performing country and to the total mean. This figure was drawn using Microsoft Office 2013 (Microsoft Corp., Redmond, WA, USA).

#### The Pearson correlation between the indicators and the Human Development Index

To investigate the relationship between maternal and child health indicators and the HDI, the Pearson correlation with a significance level of 0.05 (α=0.05) was used for analysis with SPSS version 24 (IBM Corp., Armonk, NY, USA).

#### Countries under study

To facilitate comparison, the countries under study were classified into 3 groups:

Group A, Eastern Mediterranean countries: this categorization was made by the WHO using a geographical basis. This group consists of 21 countries [19].

Group B, Upper middle-income countries: for this category, countries were divided by income using data extracted from the World Bank. Iran fell into the upper middle-income group. This group included 56 countries; American Samoa was excluded from the list due to the lack of its name and data in the SDG and in universal health coverage reports [20].

Group C, Outlook Document 1,404 (2025) countries: These 24 countries, designated for comparison with Iran until 2025, have similar conditions to Iran (Table 3) [21].

In the pooled ranking, all countries were compared as a single group, so duplicated countries (those existing in more than one category, such as Iraq, Iran, etc.) were considered only once. For some countries, numbers for some indicators were not reported and therefore were not included in the ranking comparison. Indicators with the same value for two or more countries were considered to be the same rank in the results. Due to the presence of outlier data for each indicator, the median was also reported.

### Ethics statement

This research does not have an ethical code because the data source used in this study gathered from the WHO dashboard and these data considered as secondary data.

## RESULTS

### Comparison of Iran with the other countries under study

Iran’s ranking for the 11 indicators among the countries in the three previously described categories is presented in Table 4.

### Correlations between the Human Development Index and maternal and child health indicators

**Figure 2.**
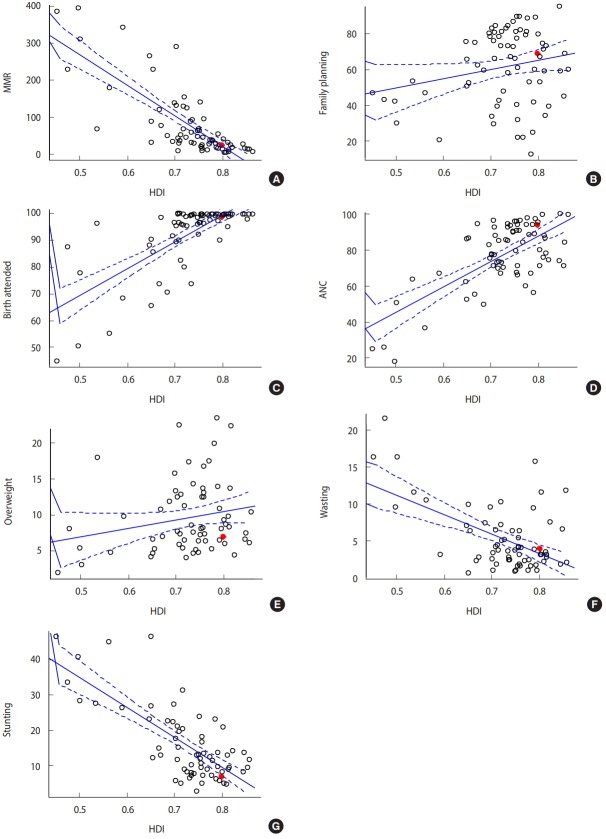
Scatter plot for Pearson correlation between indicators and Human Development Index (HDI). (A) Diphtheria-tetanus-pertussis vaccine coverage, (B) family planning, (C) birth attendance, (D) antenatal care (ANC) coverage, (E) overweight, (F) wasting, and (G) stunting. MMR, measlesmumps-rubella combined vaccine.

The results of the correlation analysis of the HDI with maternal and child health indicators are shown in Table 5 and Figure 2. In the total group, the HDI correlated significantly with the following nine indicators: maternal mortality, birth attendance, child mortality, hepatitis B incidence, diphtheria-tetanus-pertussis (DTP3) vaccine coverage, ANC coverage, care-seeking behavior of children, under-5 stunting, and under-5 wasting.

## DISCUSSION

This study found that Iran is ranked near or better than the average and median of the 75 countries analyzed. With regard to maternal mortality, Iran performed acceptably better than the median (40.00). The most direct causes of maternal mortality in Iran were bleeding during delivery (34.9%), preeclampsia (17.0%), infection (9.2%), and pulmonary embolism (7.8%) [22]. Maternal mortality has declined over the past 10 years in Iran, and it seems to be continuing to decrease due to ongoing programs. We expect that by improving deliveries and increasing the percentage of childbirths assisted by skilled personnel, maternal mortality will continue to decrease. Therefore, we recommend maintenance and improvement of population-based interventions. With regard to birth attendance by skilled personnel, Iran has 99% coverage. The target set forth by the SDG is maternal mortality below 70 deaths per 100,000 live births, which Iran appears to have already achieved. The targets for maternal mortality and for birth attendance by skilled personnel are the same. This underscores the fact that these indicators are very closely interrelated, meaning that maternal mortality is less likely in countries where the percentage of childbirth assisted by skilled personnel is higher [23]. In Iran, after the establishment of the family physician system, the number of traditional deliveries declined, and childbirth assisted by skilled personnel became more frequently welcomed and accepted [24]. We expect that, considering ongoing programs, as well as the reverse and strong correlation (in the general group) between HDI and this indicator (with HDI increasing over time), this index will improve, and the maternal mortality rate will decline as a result.

There were fewer under-5 deaths in Iran than the target set by the SDG, but the value in Iran is close to the median of the countries surveyed in this study (16.0 deaths per 1,000 live births). This value is somewhat better than the total mean, which is 23.2 deaths, although the range of values for this indicator spans from 3.5 deaths to 127.0 deaths. Between 1990 and 2015, Iran experienced a 70% reduction in the child death rate, which not only met the MDG target, but also was one of the best performances in the Middle East and North Africa over the past four decades [7,18]. The World Bank is now reporting an indicator termed Human Capital Index that some countries are opting to use instead of HDI. One of the main components of this index is the death of children under the age of 5 years. In 2018, this assessment showed that Iran ranks 70th in the world for this indicator [25,26]. Therefore, the SDGs have led to children’s health once again being re-emphasized, and the index of child death is considered one of the most important indicators of a society’s development.

Iran’s best result was for the incidence of hepatitis B; the prevalence of HBsAg antigens among children under 5 years old in Iran was found to be 0.02%. The countries surveyed displayed a range of 0.02% to 10.54% with a median of 0.37% for this indicator. Iran has achieved the goal set forth as part of the SDGs and is the leading country in the region in fighting hepatitis B infections. This achievement seems to be largely owed to the general infant vaccination program for hepatitis B, which was added to the country’s national vaccination program in 1993 [27]. In this program, children receive the hepatitis B vaccine at birth, 2 months, 4 months, and 6 months of age. Adults with high-risk occupations, such as health care workers, can also receive booster doses of the vaccine.

Iran has a 99% result for DTP3 vaccine coverage, which is among the best for countries in the region. Given the existence of regular, appropriate vaccination programs in Iran’s health systems, this trend is expected to increase. However, as seen in some other countries, the resistance to vaccination is worryingly increasing for reasons that have been described [28]. As part of Iran’s national vaccination program, children receive five doses of the DTP3 vaccine (at 2 months, 4 months, 6 months, and 18 months of age and again at age 6 years). By improving education and facilities and by increasing the HDI, vaccine coverage can be expected to reach almost 100%. Following the implementation of health system reform in Iran, the 2014 DTP vaccine has been replaced by the pentavalent vaccine; therefore, the pentavalent vaccine has been gradually included in the nationwide program in recent years.

The rate of ANC coverage (consisting of four visits or more) in Iran is 94%. This number should be compared with the mean (76.2%), the range (6.3% to 100%), and the median (79.5%) to determine whether Iran’s rate is acceptable for this indicator. Current plans for pregnancy and child delivery care in Iran include nine sessions from pre-pregnancy to the end of pregnancy: 1 visit during pre-pregnancy, 1 visit during weeks 6-10, 1 visit during weeks 16-20, 1 visit during weeks 26-30, 2 visits during weeks 31-37, and 3 visits during weeks 38-40. In this program, several items are defined for every visit regarding examinations that should be performed and values that should be recorded to take care of the health of the mother and fetus. By educating the public and thus increasing coverage for ANC while simultaneously increasing the quality of care, we expect that in addition to increasing the ANC index, maternal mortality rate, child mortality rate, and skilled birth attendance rate, pregnancy outcomes and infant-related indicators will improve.

The proportion of care-seeking behavior for suspected pediatric pneumonia patients in Iran was found to be 75.9%. This number is closer to the total median (73.8%) than the total average (70.5%). Of the countries studied, Djibouti (94.4%) had the best coverage rate. In Iran, all primary health care visits for children under-5 are free of any charges, and pneumonia care is integrated into this program. Therefore, this indicator represents the effective coverage of the health services provided as part of primary health care [29]. Regular programs must be designed and implemented to improve this index in Iran.

The prevalence of overweight in children under the age of 5 years was 6.9% in Iran. In the primary health care system of Iran, there are plans to record the height and weight of children from birth to maturity and even afterwards in health centers, as well as to conduct follow-up programs. For children in Iran’s health care system, weight gain between the ages of 0 and 5 years old is recorded using growth charts recommended by the WHO. Any deviation from the normal range, which is different for boys and girls, is considered either overweight or wasting. The prevalence of wasting among Iranian children under 5 was 4.0%. Iran is close to the median (3.7%) and is still far from reaching the SDG of the elimination of any malnutrition. In Iran, supplementation with multivitamins or vitamins A and D with iron for children under 5 years starts at birth and continues regularly afterwards. Due to ongoing programs, as well as increasing awareness of the importance of nutrition in growing children and the reverse correlation of this indicator with the HDI, we expect that this percentage will decrease gradually in Iran. Finally, the prevalence of stunting in Iran was 6.8%. Iran’s performance is relatively good compared to the median (12.7%), but improvement is still required to achieve the related SDG.

Lack of access to values for certain indicators in several countries, as well as the use of outdated data when no newer data were available, may have distorted the comparisons in this study. Another important limitation is the ecological fallacy, a bias that comes with ecological studies. In this context, when interpreting the results of the Pearson analysis between the HDI and the indicators, it should be noted that inductive inferences may be incorrect.

Iran has clearly attained good results with regard to maternal mortality, family planning, skilled birth attendance, child mortality, hepatitis B incidence, DTP3 vaccination coverage, and ANC and the country has proper plans and goals related to these areas. To achieve the SDGs, Iran needs better programs and improved performance with regard to child wasting and overweight, child stunting, and caregiving to children suspected to have pneumonia. In Iran, all of these services are provided free of charge at the level of primary health care, and the results compared to those of other countries show that these services are appropriately provided. However, given the need for effective coverage of health services that reaches the goals set as part of the SDGs, Iran must maintain focus on this agenda.

## Figures and Tables

**Table 1.** Name, definition, and goal of each indicator (WHO, 2017, 2018)

Subject	Indicator	Target for 2030 or definition	Year
Maternal mortality	3.1.1: Maternal mortality ratio	3.1: Reduce the global maternal mortality ratio to less than 70 per 100,000 live births	2015
Skilled birth attendance	3.1.2: Proportion of births attended by skilled health personnel	3.1: Reduce the global maternal mortality ratio to less than 70 per 100,000 live births	2002-2016
Child mortality	3.2.1: Under-five mortality rate	3.2: End preventable deaths of newborns and children under 5 yr of age, with all countries aiming to reduce neonatal mortality to at least as low as 12 per 1,000 live births and under-five mortality to at least as low as 25 per 1,000 live births	2017
	3.2.2: Neonatal mortality rate		
Hepatitis B incidence	3.3.4: Hepatitis B incidence per 100,000 population	3.3: By 2030, end the epidemics of AIDS, tuberculosis, malaria, and neglected tropical diseases and combat hepatitis, waterborne diseases, and other communicable diseases	2015
Family planning	3.7.1: Proportion of women of reproductive age (aged 15-49 yr) who have their need for family planning satisfied with modern methods	3.7: Ensure universal access to sexual and reproductive health care services, including for family planning, information and education, and the integration of reproductive health into national strategies and programs	2007-2016
Child immunization	One-yr old children who have received 3 doses of DTP3 (%)	DTP3, which is identical to coverage with the pentavalent vaccine in most countries, is an indicator of a routine infant immunization system; However, several other vaccines, such as for those measles (second dose), pneumococcal pneumonia, and rotavirus diarrhea, typically have lower coverage, and the fraction of children receiving all vaccines in a national schedule is typically much lower (although not possible to measure directly with existing data systems in most countries); This indicator could be replaced with a second dose of measles vaccine, following the recent recommendation of the Strategic Advisory Group of Experts on Immunization	2017
Pregnancy and delivery care	ANC, four or more visits 4 (%)	No. of ANC visits captures contact with the health system but it does not capture quality of care received and may not lead to improved mortality outcomes	2002-2016
Child treatment	Care-seeking behavior for children with suspected pneumonia (%)	Pneumonia is a leading cause of child illness and death; Suspected pneumonia is determined based on a series of survey questions about illnesses in the past two weeks, which may include mild respiratory illnesses; The indicator does not currently capture the quality of care received, as parental recall of treatment specifics tends to be poor	1993-2016
Stunting among children	2.2.1: Prevalence of stunting (height for age <-2 SD from the median of the WHO Child Growth Standards) among children under 5 yr of age	2.2: By 2030, end all forms of malnutrition, including achieving, by 2025, the internationally agreed targets on stunting and wasting in children under 5 yr of age, and address the nutritional needs of adolescent girls, pregnant and lactating women and older persons	1995-2016
Wasting and overweight among children	2.2.2: Prevalence of malnutrition (weight for height >+2 SD or <-2 SD from the median of the WHO Child Growth Standards) among children under 5 yr of age, by type (wasting and overweight)	2.2: By 2030, end all forms of malnutrition, including achieving, by 2025, the internationally agreed targets on stunting and wasting in children under 5 yr of age, and address the nutritional needs of adolescent girls, pregnant and lactating women and older persons	1989-2016

WHO, World Health Organization; AIDS, acquired immune deficiency syndrome; DTP3, diphtheria-tetanus-pertussis; ANC, antenatal care; SD, standard deviation.

**Table 2.** Comparison of indicators between Iran and the other countries in the 3 groups

Indicator	World mean (World Bank)	Total median	Weighted mean			Iran	Best	Worst
			Upper middle-income	Eastern Mediterranean	Outlook Document			
Maternal mortality	216.00	40.00	36.90	161.49	121.59	25.00	Belarus and Kuwait (4.00)	Somalia (723.00)
Skilled birth attendance	80.01	97.85	98.43	70.62	78.24	99.00	Turkmenistan and Uzbekistan (100.00)	Somalia (9.40)
Child mortality	40.80	16.00	12.11	23.41	40.23	14.90	Montenegro (3.50)	Somalia (127.00)
Hepatitis B incidence	-	0.37	0.61	0.81	1.23	0.02	Iran (0.02)	Somalia (10.54)
Family planning	-	63.85	85.73	50.27	53.95	68.60	China (95.00)	Albania (12.90)
DTP3 coverage	85.58	94.00	95.30	89.70	84.55	99.00	16 countries (99.00)	Equatorial Guinea (25.00)
Antenatal care	75.00	79.50	79.55	68.18	61.56	94.00	Bahrain (100.00)	Somalia (6.30)
Care-seeking behavior for children	60.00	73.80	74.07	64.32	62.73	75.90	Djibouti (94.40)	Somalia (13.00)
Stunting	22.20	12.70	9.99	17.62	25.97	6.80	St. Lucia (2.50)	Yemen (46.50)
Overweight	5.60	7.85	7.35	7.72	7.43	6.90	Yemen (2.00)	Albania (23.40)
Wasting	7.50	3.70	2.35	7.68	7.99	4.00	Guatemala (0.70)	Djibouti (21.50)

DTP3, diphtheria-tetanus-pertussis.

**Table 3.** Classification of countries analyzed in this study

Country	Eastern mediterranean	Upper-middle-income	Outlook Document	Country	Eastern mediterranean	Upper-middle-income	Outlook Document
Afghanistan	O	-	O	Kuwait	O	-	O
Albania	-	O	-	Lebanon	O	O	O
United Arab Emirates	O	-	O	Libya	O	O	-
Armenia	-	O	O	St. Lucia	-	O	-
Azerbaijan	-	O	O	Morocco	O	-	-
Bulgaria	-	O	-	Maldives	-	O	-
Bahrain	O	-	O	Mexico	-	O	-
Bosnia and Herzegovina	-	O	-	Marshall Islands	-	O	-
Belarus	-	O	-	Macedonia	-	O	-
Belize	-	O	-	Montenegro	-	O	-
Brazil	-	O	-	Mauritius	-	O	-
Botswana	-	O	-	Malaysia	-	O	-
China	-	O	-	Namibia	-	O	-
Colombia	-	O	-	Nauru	-	O	-
Costa Rica	-	O	-	Oman	O	-	O
Cuba	-	O	-	Pakistan	O	-	O
Djibouti	O	-	-	Peru	-	O	-
Dominica	-	O	-	Paraguay	-	O	-
Dominican Republic	-	O	-	Qatar	O	-	O
Algeria	-	O	-	Romania	-	O	-
Ecuador	-	O	-	Russia	-	O	-
Egypt	O	-	-	Saudi Arabia	O	-	O
Fiji	-	O	-	Sudan	O	-	O
Gabon	-	O	-	Somalia	O	-	-
Georgia	-	-	O	Serbia	-	O	-
Equatorial Guinea	-	O	-	Suriname	-	O	-
Grenada	-	O	-	Syria	O	-	O
Guatemala	-	O	-	Thailand	-	O	-
Guyana	-	O	-	Tajikistan	-	-	O
Iran	O	O	O	Turkmenistan	-	O	O
Iraq	O	O	O	Tonga	-	O	-
Jamaica	-	O	-	Tunisia	O	-	-
Jordan	O	O	O	Turkey	-	O	O
Kazakhstan	-	O	O	Tuvalu	-	O	-
Kyrgyzstan	-	-	O	Total	21	56	24

**Table 4.** Comparison of Iran’s rank for relevant indicators with the other countries analyzed

Indicator	Rank		
	Eastern Mediterranean countries (out of n countries)	Outlook Document countries (out of n countries)	Upper-middle income countries (out of n countries)
Maternal mortality	8 (21)	9 (24)	11 (51)
Birth attendance by skilled personnel	4 (20)	7 (23)	9 (54)
Child mortality	9 (21)	12 (24)	24 (55)
Hepatitis B incidence	1 (21)	1 (24)	1 (55)
Immunization coverage	1 (21)	1 (24)	1 (55)
Care-seeking behavior for children with an acute respiratory infection	9 (21)	8 (24)	19 (52)
Family planning	5 (21)	5 (24)	27 (54)
Antenatal care	4 (21)	8 (24)	10 (55)
Stunting	2 (20)	2 (23)	9 (50)
Wasting	6 (20)	8 (23)	21 (50)
Overweight	8 (20)	10 (23)	16 (50)

**Table 5.** Correlation of the Human Development Index with each indicator (α=0.05)

Indicator	Total		Eastern Mediterranean		Prospective Outlook		Upper-middle income	
	p-value	R	p-value	R	p-value	R	p-value	R
Maternal mortality ratio	<0.001	-0.787	<0.001	-0.861	<0.001	-0.852	<0.001	-0.728
Birth attendance	<0.001	0.721	<0.001	0.715	<0.001	0.784	<0.001	0.664
Child mortality	<0.001	-0.808	<0.001	-0.861	<0.001	-0.838	<0.001	-0.781
Hepatitis B incidence	<0.001	-0.419	0.002	-0.639	<0.001	-0.710	<0.001	-0.484
DTP3 vaccine coverage	<0.001	0.573	0.001	0.705	<0.001	0.681	<0.001	0.550
Antenatal care coverage	<0.001	0.683	<0.001	0.850	<0.001	0.770	0.039	0.248
Care-seeking	0.003	0.346	0.049	0.445	0.016	0.485	0.045	0.282
Family planning	0.059	0.225	0.061	0.426	0.078	0.367	0.359	0.130
Overweight under 5	0.095	0.209	0.494	0.167	0.212	0.271	0.203	0.189
Stunting under 5	<0.001	-0.734	<0.001	-0.874	<0.001	-0.872	<0.001	-0.505
Wasting under 5	<0.001	-0.541	0.001	-0.715	<0.001	-0.706	0.836	-0.031

R, Pearson correlation coefficient; DTP3, diphtheria-tetanus-pertussis.
